# The Schmidt Treatment for Parturient Paralysis

**Published:** 1901-09

**Authors:** John J. Repp

**Affiliations:** Veterinarian to the Experiment Station, Ames, Ia.


### THE JOURNAL

OF

### COMPARATIVE MEDICINE AND VETERINARY ARCHIVES.

VOL. XXII. *SEPTEMBER, 1901.* No. 9.

### THE SCHMIDT TREATMENT FOR PARTURIENT PARALYSIS.

BY JOHN J. REPP, V.M.D.,

VETERINARIAN TO THE EXPERIMENT STATION, AMES, IA.

THIS disease is also known by the names parturient paresis, paresis of parturition, parturient fever, milk fever. The word "fever" in connection with the terminology of this disease is not very appropriate, because in the majority of cases fever is not present, but the animal has a subnormal temperature. The term milk fever is very misleading and indefinite, as it is also used by the laity to designate other diseases, such as parturient septicæmia and the various forms of mammitis. Parturient paralysis must be clearly differentiated from parturient septicæmia, which is a disease of an entirely different character and which may occur in any of the domestic species, whereas parturient paralysis occurs only in the cow.

*History.* Although this history of the disease is rather obscure, it is probably quite an old one.

*Distribution.* Parturient paralysis occurs wherever milk cows are kept. It is more prevalent in dairy districts, because it is the heavy milking strains of cows that are most subject to the disease. As far as I am able to learn, it is quite prevalent in certain sections of our own State, while in other sections it rarely occurs. In the same locality more cases occur in some years than in others. This irregularity of distribution does not appear to throw any light upon the cause of the disease.

*Cause.* No definite cause can be assigned for this disease. We cannot do more than theorize upon this point. The scope of this article will not permit the discussion of these various theories, therefore it shall be limited to a brief consideration of the theory which more especially concerns the present discussion, namely, that of Veterinarian J. Schmidt, of Kolding, Denmark. Schmidt's theory is that parturient paralysis is caused by the evolution in the mammary gland of a poisonous substance through the over-activity of the epithelial cells of this gland excited by the determination to the udder after birth of large quantities of blood which was supplied to the uterus and the fœtus before birth, but which now goes to the udder because of the natural demand for milk secretion. This poisonous substance being carried in the circulation to various parts of the body, brings on the symptoms which characterize the disease. It is well recognized that living cells may, under certain circumstances, produce poisonous substances. Schmidt's theory, therefore, is in accord with an established principle.

*Pathogenesis, or Generation, of the Disease.* Parturient paralysis, as a rule, occurs in cows which give a heavy flow of milk and which are in a high state of nutrition. It may develop at any age, but is extremely rare in cows before they have reached adult age and have given birth to several calves. It is also rare in old cows. It occurs, then, in cows which are of middle age and in the full height of their activity as milk producers. The disease attacks the cow after she has given birth to a calf, usually within twenty-four hours thereafter, but in some cases not until a week or even a month after parturition. In a few cases the disease has its inception a short time before parturition. Cows which are stabled and deprived of exercise are said to be more prone to the disease than those which are permitted to exercise at will. There are many exceptions to this statement, although it is the usual teaching. Further observation may show that it is not correct. The report given in this bulletin indicates that in Iowa more cows take this disease while at pasture than in any other circumstance. This doubtless arises from the fact that in Iowa cows are given more freedom than is customary in older dairy States. The disease may arise at any time in the year, but, on account of the fact that springtime is pre-eminently the calving season, most cases originate at this season.

*Morbid Anatomy.* The morbid alterations are limited and variable, and offer nothing characteristic. The blood is irregularly distributed, a condition which probably indicates marked vaso-motor disturbance resulting from the profound interference with the nervous functions which accompanies the disease. The abdominal organs are usually filled with blood. The brain may be anæmic, œdematous, easily torn, and yellowish in color. In other cases it shows hyperæmia of the meninges and of the brain substance.

*Symptoms.* The disease usually appears within twenty-four to forty-eight hours after parturition. In extreme cases it may not occur until two months or even six months after parturition. It may rarely occur before birth. It usually follows an easy birth. At the onset of the disease the cow manifests some uneasiness; she moves about in a restless manner, stamps, strikes the abdomen with hind legs, perhaps bellows, grinds the teeth, and may have spasms of groups of muscles or even a general convulsion. After this period, which may be unnoticed, the symptoms of paralysis come on. The cow shows weakness, staggers, and at last falls. As the paralysis advances she stretches on the ground, lying on her side usually with the neck bent to one side so as to bring the nose into the flank or the costal region. This is the characteristic position of parturient paralysis. If the head is brought into the normal position, it at once returns to the unnatural position in which it was found. The animal is in a state of partial or complete unconsciousness, does not respond to blows or calls and takes no note of her surroundings. The eye is dull and not sensitive to the finger touch, sunken, pupil dilated, and the upper lid is drooping; the tongue is paralyzed, saliva runs from the mouth, the pharynx and œsophagus have lost the power of motion, so that the animal is unable to swallow; the peristalsis of the stomachs and intestines is in abeyance, and as a result digestion is arrested, fermentation sets in, and the animal becomes tympanitic; the contents of the rectum and colon are hard and dry, and may be covered with mucus or blood, urination is suspended; the os uteri is almost invariably dilated if the disease occurs within a day of parturition; pulse small, often imperceptible, 60 to 120 per minute; temperature usually normal or below normal, may be as low as 95° F., in some cases may be as high as 105° F. Such a high temperature probably does not occur in a case of pure parturient paralysis, but only when there is a complication of parturient septicæmia. The extremities are cold. The after-birth is sometimes retained. There may be accompanying prolapse of the uterus.

*Course.* Without treatment, and, indeed, with most kinds of treatment which have been applied in the past, the disease usually runs rapidly to a fatal issue. It lasts two to three days, and in some cases longer, the condition gradually becoming more and more aggravated. Death results from sudden failure of the heart or brain, and is often preceded by profuse diarrhœa. In milder cases the cow may linger as long as two to four weeks and then die of pneumonia, which results from the inhalation, or introduction through attempts at medication, of foreign substances into the lungs during the period of paralysis of the pharynx and œsophagus. If recovery occurs, the animal is entirely well in two to five days. In rare cases paralysis of the hind parts may persist for a long while.

*Diagnosis.* This is made by a study of the history and symptoms. It is comparatively easy.

*Differential Diagnosis.* It must be distinguished from ante-partum paralysis, broken-back, parturient septicæmia; but one familiar with the character of these diseases will find no difficulty in making this differentiation.

*Treatment.* This may be considered under two distinct subdivisions, viz.: preventive treatment and curative treatment.

(*a*) Preventive treatment: By considering what has been said under the head of "generation of the disease" one can easily infer what measures should be adopted to prevent the disease. Cows in the later stage of gestation should be fed moderately, grain especially being given sparingly or entirely withheld; the animals should be given an opportunity to take plenty of exercise; the bowels should be kept in good condition by the administration of such salines as magnesium sulphate, sodium chloride, and sodium bicarbonate. The afterbirth should be removed soon after parturition and several uterine douches administered.

(*b*) Curative treatment: The object of this bulletin being to report the results of the treatment of parturient paralysis by the method first suggested and recommended by Schmidt-Kolding, the discussion shall be confined to that treatment to the exclusion of the many others that have at various times been brought forward and tried, but which give but little encouragement for the continuance of their use.

Schmidt does not claim that his method of treatment disposes bodily of the morbid condition, but that it does measurably assist nature in her efforts to restore the animal to the normal physiological state. It is well known that after the beginning of the attack the animal, if left to herself, rapidly grows worse until the crisis of the disease is reached, at which time death occurs or convalescence begins, usually the former. It has been observed, however, that if the treatment is applied within a few hours after the inception of the disease its progress is modified in such a way that convalescence at once begins, as a rule, and the animal hastily recovers her health, usually within twelve hours, although in extreme cases it may be as late as forty-eight hours. Following is an outline of the plan of treatment of parturient paralysis suggested by Schmidt. The operator should disinfect his hands and the udder and teats of the cow by washing with a 5 per cent. solution of carbolic acid or creolin, or a 1½ per cent. solution of lysol or trikresol. The apparatus needed for the treatment consists in a small glass funnel, a rubber hose three feet long and one-eighth inch in calibre into which the funnel fits, and an ordinary milking tube over which the rubber hose fits. This apparatus should be sterilized immediately before it is used by boiling or soaking in such a solution as recommended for washing the udder. Dissolve from two to two and one-half drachms of potassium iodide—the size of the dose depending upon the size of the cow and the character of the attack—in about one quart of clean water previously boiled to sterilize it, and allow the solution to cool to a little above body temperature, or 40° C. or 104° F. The temperature may be determined with the clinical thermometer. Withdraw all the colostrum or milk from the udder. Then insert the milking tube, with hose and funnel attached, into one of the teats, elevate the funnel about two feet above the teat and slowly pour in one-fourth of the solution, allowing the funnel and hose to become empty several times during the process in order to permit the entrance of a liberal quantity of air. Repeat this infusion with the other three-quarters of the udder. After all is introduced knead the udder carefully so as to cause the solution to permeate the ducts and acini as much as possible.

As the condition of the cow is usually such as to indicate additional treatment, the veterinarian should not be content with simply the infusion of the potassium iodide solution, but should resort to other measures which promise assistance.

As the cow is usually unable to urinate the bladder will be found filled with urine. This should be removed with the catheter and its removal accomplished at intervals until the recovery of the cow renders this procedure no longer needful.

It may be advisable that catharsis be brought about. As the cow is usually unable to swallow, it is dangerous to attempt to give medicines by the mouth. This may be done if assurance that the cow can swallow is obtained. Some have given medicines successfully through a probang inserted into the stomach. This plan is feasible. Schmidt says that he usually resorted to an aloe powder. If this is done one ounce to one and one-half ounces of aloes may be given. It would seem preferable to give the aloes in a bolus, capsule, or drench. Some have given linseed oil or Epsom salts. If the animal cannot swallow and a probang is not at hand, one may administer one and one-half to two grains of physostigmine salicylate subcutaneously, repeating the dose in about three hours if purgation is not produced. Rectal injections should be given at short intervals in order to get rid of the accumulation of hard, dry feces in the rectum. These injections may be of linseed oil, cottonseed oil, or warm soap solution. Schmidt recommends, also, enemata of sodium chloride solution. Meanwhile the cow should be kept propped up on the sternum by means of bags of straw or pieces of wood. If the temperature is below normal, as it usually is, the cow should be thickly clothed with blankets and straw heaped up about her. Schmidt used powdered digitalis given by the mouth when the heart was rapid and weak. It would seem much better in every way to give the tincture of digitalis subcutaneously. He has also resorted to subcutaneous injections of camphor and caffeine. This is good treatment. If the cow does not show marked improvement within eight hours the potassium iodide infusion may be repeated. Schmidt has found that as high as six drachms may be given into the udder without harm to the cow. Schmidt, in his first report, made in 1898, recorded fifty cases treated for parturient paralysis by this method with but two deaths from the disease. There were, however, only forty-six recoveries, as two cows were slaughtered for beef during the first day of convalescence. A short time later a report was made by Jensen showing that in Denmark up to that time sixty-five veterinarians of that country had treated 412 cases by the Schmidt method, 90 per cent. of which recovered. These results seem to indicate that this was the treatment *par excellence* for this disease. It still remained to secure the introduction of this treatment into the United States and to determine what results could be obtained by our veterinarians through this application. For the purpose of assisting in the introduction of the Schmidt treatment for parturient paralysis into Iowa and of determining what the results of this treatment would be in the hands of the veterinarians of this country, in September, 1899, I addressed a circular letter to each of the 150 graduate veterinarians in Iowa requesting their cooperation in the work of this research by applying the treatment in their practice and reporting their results to me. Ninety of these replied, signifying their willingness to use the treatment. I should say that a few of these had already put the treatment into use in their practice. These, however, agreed to report their subsequent results to me. Accordingly a circular of instructions, an infusion apparatus, two ounces of potassium iodide, and ten special blanks for report of cases treated were forwarded to each veterinarian who had consented to co-operate. In November, 1900, a letter was issued to each of these veterinarians requesting that the reports they had collected be sent in. Up to this time I have received replies from thirty-three veterinarians. In all 166 cases were reported ; of these 166, 119 resulted in recovery, while 47 were fatal. Of the fatal cases, in 8 of the cows death may be traced to some complication, such as prolapse of the uterus, foreign body, pneumonia, etc. In these cases the Schmidt treatment cannot be saïd to have failed, for it is not in any way intended that it shall be able to overcome such accidental conditions. If the cow has recovered from her condition of paralysis as a result of the Schmidt treatment far enough to be out of danger from that source and to promise recovery, but later falls a victim to some complication that is in no measure a part of parturient paralysis, but only a result of that disease, it may with justice be said that the Schmidt treatment was a success so far as the malady against which it was directed is concerned. Looking at the reports from this generous point of view, in 127 cases out of 166, or 76.5 per cent., the Schmidt treatment was successful so far as the parturient paralysis was concerned.

I regret that the limit of this article forbids the presentation of a full report of each case, and that it must be confined to the following tabulated report, which gives a summary of the important features of these reports :

1. Breed—

| | | | |
|---|---|---|---|
| Shorthorn | *31 | Galloway | 1 |
| Jersey | 17 | Polled Angus | 1 |
| Holstein | 4 | Devon | 1 |
| Hereford | 3 | Grade | 42 |
| Not stated | 66 | | |

2. Age—

| | | | |
|---|---|---|---|
| Four years | 2 | Nine years | 10 |
| Five years | 8 | Ten years | 8 |
| Six years | 19 | Twelve years | 5 |
| Seven years | 24 | Thirteen years | 1 |
| Eight years | 26 | Not stated | 63 |

3. Condition—

| | | | |
|---|---|---|---|
| Fat | 33 | Thin | 2 |
| Fair | 57 | Not stated | 74 |

* The figures represent the number of cases.

4. Quantity of milk given—

| | | | |
|---|---|---|---|
| Very large | 41 | Small | 6 |
| Large | 24 | Not stated | 95 |

5. Care and feeding before parturition—

| | | | |
|---|---|---|---|
| Fed lightly | 3 | Pasture, other feed | 19 |
| Stabled, fed liberally | 14 | Not stated | 78 |
| Pasture only | 52 | | |

6. Had parturient paralysis before—

| | | | |
|---|---|---|---|
| Once | 4 | Twice | 2 |

7. Character of parturition—

| | | | |
|---|---|---|---|
| Normal | 80 | Not stated | 83 |
| Difficult | 3 | | |

8. Number of calves borne by each cow—

| | | | |
|---|---|---|---|
| One calf | 1 | Six calves | 14 |
| Two calves | 2 | Seven calves | 9 |
| Three calves | 13 | Eight calves | 2 |
| Four calves | 19 | Nine calves | 1 |
| Five calves | 19 | Not stated | 88 |

9. After-birth retained—8

10. Prolapsus uteri—2

11. State of os uteri during attack—

| | | | |
|---|---|---|---|
| Relaxed | 28 | Not stated | 130 |
| Contracted | 8 | | |

12. Disease appeared before parturition . . . . . . . 1

13. Length of time between parturition and first symptoms of disease—

| | | | |
|---|---|---|---|
| One hour | 2 | Twenty hours | 9 |
| Two hours | 1 | Twenty-four hours | 19 |
| Three hours | 2 | Thirty hours | 8 |
| Six hours | 2 | Thirty-six hours | 9 |
| Eight hours | 7 | Two days | 5 |
| Ten hours | 6 | Three days | 7 |
| Twelve hours | 9 | Five days | 1 |
| Fourteen hours | 7 | Eight days | 1 |
| Sixteen hours | 5 | Not stated | 59 |
| Eighteen hours | 7 | | |

Time between inception of disease and beginning of Schmidt treatment: One to twenty-four hours.

14. Number of times infusion of potassium iodide was repeated—

| | | | |
|---|---|---|---|
| Once | 22 | Twice | 2 |

15. Time elapsing between application of Schmidt treatment and time the cow was able to stand—

| | | | |
|---|---|---|---|
| One hour | 1 | Sixteen hours | 6 |
| Two hours | 1 | Twenty hours | 5 |
| Four hours | 1 | Twenty-four hours | 4 |
| Five hours | 1 | Thirty hours | 5 |
| Six hours | 11 | Two days | 6 |
| Eight hours | 10 | Three days | 2 |
| Ten hours | 5 | Four days | 3 |
| Twelve hours | 5 | Not stated | 94 |
| Fourteen hours | 6 | | |

16. Number of relapses 4 (fatal).

17. Number of cases of pneumonia 6 (fatal).

18. Quantity of milk secreted during first five days after treatment—

| | | | |
|---|---|---|---|
| Small | 26 | Not stated | 137 |
| Normal | 3 | | |

19. Number of cases in which Schmidt treatment was followed by mastitis, 7.

That in many cases in the above table certain features are put down as "not stated" is in large measure accounted for by the fact that only 110 of the reports were made on the blanks which I sent to the practitioners, the rest being made in the form of a brief summary in a letter. Of these reports made on the blanks only a few gave in detail all of the particulars called for by the questions. I infer, however, that the features set forth in the table may be taken as a fair representation of all the cases treated.

The table shows that most of the cows affected were grades. I may add that most of these were shorthorn grades. Next in point of numbers is the shorthorn breed. It should not be inferred from this that the shorthorn breed is more susceptible, for it is probable that the order of numbers affected among the various breeds is in close proportion with the number of cows of these breeds in the State.

It will be seen by reference to the table that cows affected were chiefly between the ages of six and nine years and at the birth of the third to the sixth calf. This is in accordance with observations elsewhere. Only one of these cases occurred in the primipara and only two in the secundipara.

Nearly all the animals were reported either fat or very fat. Only two cows affected by the disease are reported as thin in flesh. This also is in accord with the usual observation.

The table is in a measure contradictory to some of the current teachings on this subject in respect to the care and feeding of the animal prior to parturition. In the majority of the cases in which this feature was reported upon the cows were at pasture and had no other food. They also had plenty of exercise. Only fourteen were stabled and fed liberally, conditions generally thought to predispose to this disease. Only nineteen were at pasture and received in addition other food. That most of the cows took the disease while at pasture and not receiving any other food may arise from the fact that this is the way in which most cows are kept in Iowa during the pasture season. We cannot conclude that pasture alone may not constitute a liberal diet to a cow with good appetite and digestion, although it is generally considered that if the grass diet is not supplemented with grain ration the cow is reasonably safe from an attack of parturient paralysis. The above figures would indicate that the conclusion in this respect needs some revision. It is encouraging to note that only three cases occurred in cows fed lightly with a view to preventing the disease.

It is worthy of note as being in accord with the usual teaching that nearly all the cows were large or very large milkers.

In nearly every case the parturient act was accomplished in a normal manner. In only three cases did parturient paralysis develop after a difficult parturition. This, however, may be in the same ratio as the normal to the difficult births not followed by the disease. Were this so it would not damage Schmidt's theory of the etiology of the disease, for in order to prove his theory it is not at all necessary to show that the disease followed an easy birth, although Schmidt cites this circumstance in support of his theory.

The table would indicate that one may look for this disease most commonly within the first twenty-four hours after the calf is born. In one case, however, it had its onset before parturition; in others, after the first day.

The table shows that the infusion of potassium iodide into the udder was repeated in the course of eight to twelve hours in twenty-two cases, and was repeated twice in two cases. Of the twenty-two cases which received two doses each, seven were fatal, two having had relapses and one pneumonia. There is no means of learning from the reports just what the second or the third dose contributed toward the cure of the disease, but as all these cases were severe ones and the repetition of the dose was strongly indicated, it may be inferred that it is good practice to repeat the dose in eight to twelve hours, provided the cow has not then responded to previous treatment. I think it may be safely concluded that twenty or even thirty grammes of potassium iodide injected into the udder within twenty-four hours will not do harm, but, on the other hand, may contribute largely toward the cure of the case. It would not be advisable, however, to repeat the dose if the cow has responded to the first one.

It appears from the table that one may expect the cow to have pretty well recovered from her attack within twelve hours after the treatment is administered, although some cases do not yield so soon. A relapse may be expected at any time, and it would seem that this renders the prognosis very grave.

Pneumonia is a very grave complication. None of the six cows in which this complication arose recovered. This leads to an inquiry into the cause of the pneumonia. It may be due to dust or particles inhaled during the attack, but it is most likely set up in most cases by the entrance into the lungs of drenches designed for the stomach, but failing to reach their destination on account of the inability to swallow. This would lead me to recommend that no medicines be given by the mouth while the animal is unable to swallow, unless given by means of the probang. The heart stimulants, such as strychnine, atropine, caffeine, tincture of digitalis, can readily be given hypodermically, as can also the cathartic in the shape of a dose of physostigmine. One would judge, however, that, since physostigmine is such a powerful motor depressant, it would be contraindicated in this disease, yet it has been given with favorable results. For my own part I should not use it, and cannot recommend its use. It is not manifest upon examining the theory of the etiology of the disease and the state of the alimentary canal that a cathartic is strongly indicated. The fecal matter is usually normal except in the lower part of the large intestine and the rectum, from which places it may be dislodged by enemata and by manual means. Would it not be well, then, to withhold the purgative at least until the cow has regained the power to swallow? I believe, withal, it is good practice to give a cathartic whenever the cow is able to swallow.

One practitioner who has treated a large number of cases with very good success frequently gives nothing but the infusion of potassium iodide into the udder. There is no doubt that many cases are hurried on to a fatal termination by excessive medication. Just where to draw the line is a difficult point which each practitioner must decide in the case before him.

The report of seven cases followed by mastitis warns us that too much care cannot be exercised in sterilizing everything that comes into contact with the udder during the process of introducing the potassium iodide solution. It is certainly true that the potassium iodide alone will not give rise to this accident, which should not be charged against the treatment. It is probably the result of infection introduced into the udder by lack of adequate disinfection.

In most cases reported milk secretion was much below normal in amount for a few days after the injection of the potassium iodide, but was restored to normal in all cases in which mastitis did not develop. This is to be expected, for the treatment brings success partly because it diminishes cellular activity in the udder.

Although the reports do not show the relative value of treatment early in the disease and late in the disease, for some cows treated early promptly died, while others treated as late as twenty-four hours got well; yet it is fair to conclude that the earlier the treatment is instituted the more likely it is that the cow will recover.

In the event that the cow suffers from tympanites during the attack of parturient paralysis, resort must be had to puncture of the rumen with the trocar. Some veterinarians have administered medicine through the canula after the puncture has been made and the gases evacuated. This procedure is doubtless as useful as it is unique.

With 76.5 per cent. of cures to the credit of the Schmidt treatment in the hands of the general practitioner in Iowa, who is called upon to treat these cases under all sorts of adverse circumstances, it only remains to advise that this treatment be promptly resorted to in all cases of parturient paresis. If it is not infallible it is at least the best form of treatment for this malady that has thus far been introduced.

It is also to be expected that as veterinarians acquire more practice in its application they will be able to apply it better and with a greater degree of success.

